# Non-atherosclerotic coronary stenosis potentially triggered by recurrent coronary vasospasm: insights from optical coherence tomography, intravascular ultrasound, and near-infrared spectroscopy

**DOI:** 10.1093/ehjcr/ytaf432

**Published:** 2025-08-29

**Authors:** Keima Wayama, Kota Murai, Teruo Noguchi

**Affiliations:** Department of Cardiovascular Medicine, National Cerebral and Cardiovascular Center, 6-1, Kishibe-shinmachi, Suita, Osaka 564-8565, Japan; Department of Cardiovascular Medicine, National Cerebral and Cardiovascular Center, 6-1, Kishibe-shinmachi, Suita, Osaka 564-8565, Japan; Department of Cardiovascular Medicine, National Cerebral and Cardiovascular Center, 6-1, Kishibe-shinmachi, Suita, Osaka 564-8565, Japan

A 53-year-old man had been experiencing recurrent effort angina exclusively in the morning for two years. He had a medical history of dyslipidaemia and was a former smoker. Coronary angiography revealed intermediate stenosis in the left circumflex artery (LCX) (*Panel A*, arrow). Given the suspicion of vasospastic angina, provocation testing with acetylcholine was performed. Angiography following the administration of 20 μg of acetylcholine demonstrated total occlusion at the LCX stenosis (*Panel B*, arrowhead), accompanied by ipsilateral collateral circulation, suggesting repeated episodes of vasospasm (*Panel C*, arrowheads) (see Supplementary material online, *Video S1*). The occlusion was relieved after nitroglycerine injection; however, the intermediate stenosis persisted (*Panel D*, arrow). As the subsequently measured fractional flow reserve was 0.80, percutaneous coronary intervention (PCI) was performed based on prior evidence suggesting that coronary spasm occurring at sites of significant organic stenosis is associated with adverse cardiovascular outcomes, although the clinical benefit of PCI in such cases has not been clearly established.

**Figure:**
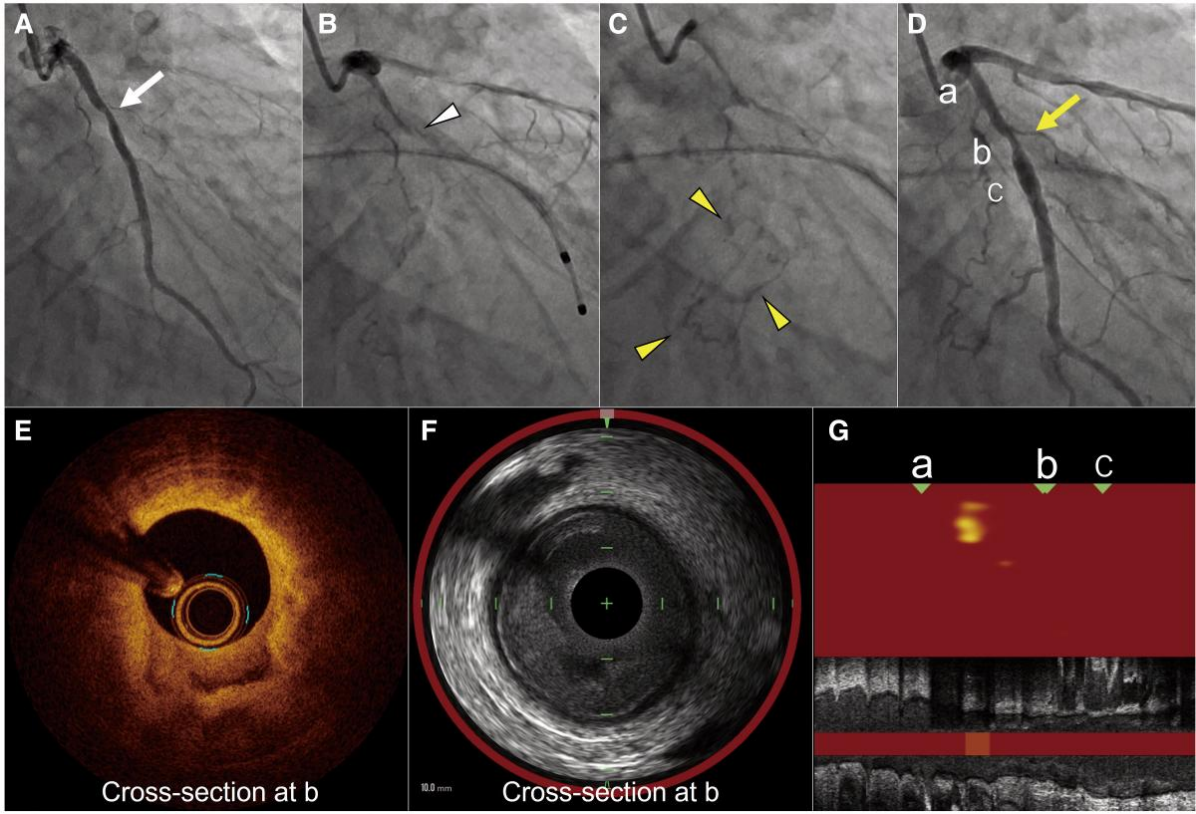


Optical coherence tomography (OCT) revealed a layered appearance with superficial areas of signal attenuation, but no apparent plaque rupture (*Panel E*, Supplementary material online, *Video S2*). Although thrombus organization within the coronary arterial wall was suspected, it remained unclear whether this was due to atherosclerotic plaque rupture or a non-atherosclerotic process such as plaque erosion. To further clarify plaque composition and lipid content, additional evaluation with near-infrared spectroscopy–intravascular ultrasound (NIRS-IVUS) was performed. IVUS demonstrated a layered plaque without signal attenuation (*Panel F*). NIRS showed no yellow signals in the short-axis view, and a maximum lipid-core burden index within 4 mm at the lesion was 139, which was considered low in comparison with previous studies (*Panels F* and *G*). These findings suggested a non-atherosclerotic stenosis, potentially due to an organized thrombus caused by recurrent vasospasm. A drug-eluting stent was successfully deployed, and calcium channel blockers were prescribed, leading to symptom improvement.

Repeated episodes of vasospasm may cause endothelial injury, promote mural thrombus formation, and contribute to the progression of stenosis even in the absence of lipid-rich atherogenic features. This case highlights the potential of multimodality intravascular imaging in improving the accuracy of plaque characterization, demonstrating the progression of obstructive coronary artery disease due to untreated ischaemia in the setting of ‘INOCA’.

## Supplementary Material

ytaf432_Supplementary_Data

## Notes

**Consent:** Written informed consent was obtained from the patient for publication of this case in accordance with the COPE guidelines.

**Funding:** None.

**Data availability:** No data were generated or analysed for or in support of this paper.

